# Exploring Mechanisms of Selective Directed Forgetting

**DOI:** 10.3389/fpsyg.2017.00316

**Published:** 2017-03-03

**Authors:** Carmen Aguirre, Carlos J. Gómez-Ariza, Pilar Andrés, Giuliana Mazzoni, Ma T. Bajo

**Affiliations:** ^1^Department of Experimental Psychology, University of GranadaGranada, Spain; ^2^Research Center for Mind, Brain and Behavior, University of GranadaGranada, Spain; ^3^Department of Psychology, University of JaénJaén, Spain; ^4^Department of Psychology, University of the Balearic IslandsBalearic Islands, Spain; ^5^Research Institute on Health SciencesBalearic Islands, Spain; ^6^Department of Psychology, University of HullHull, UK

**Keywords:** episodic memory, motivated forgetting, selective directed forgetting, executive control, inhibition

## Abstract

While some studies have shown that providing a cue to selectively forget one subset of previously learned facts may cause specific forgetting of this information, little is known about the mechanisms underlying this memory phenomenon. In three experiments, we aimed to better understand the nature of the selective directed forgetting (SDF) effect. Participants studied a List 1 consisting of 18 sentences regarding two (or three) different characters and a List 2 consisting of sentences regarding an additional character. In Experiment 1, we explored the role of rehearsal as the mechanism producing SDF by examining the effect of articulatory suppression after List 1 and during List 2 presentation. In Experiments 2 and 3, we explored the role of attentional control mechanisms by introducing a concurrent updating task after List 1 and during List 2 (Experiment 2) and by manipulating the number of characters to be selectively forgotten (1 out of 3 vs. 2 out of 3). Results from the three experiments suggest that neither rehearsal nor context change seem to be the mechanisms underlying SDF, while the pattern of results is consistent with an inhibitory account. In addition, whatever the responsible mechanism is, SDF seems to rely on the available attentional resources and the demands of the task. Our results join other findings to show that SDF is a robust phenomenon and suggest boundary conditions for the effect to be observed.

## Introduction

Many everyday situations require updating information by exerting control over our memory. For example, people may sometimes provide us with wrong instructions to perform a task or they might give us an erroneous direction to find a specific location. If this happens and we realize, we should forget the wrong instructions or directions so that they do not compete with the right information we need. Usually updating of information is specific and selectively targeted to the information that is no longer relevant, so that the correct instructions and information are kept in mind to be able to successfully perform the intended task. However, while many studies have sought to investigate how people intentionally forget information in a non-selective way (e.g., Conway et al., 2000; Sahakyan and Kelley, 2002; Sahakyan and Delaney, 2003; Delaney et al., 2010; Hanslmayr et al., 2012; Abel and Bäuml, 2016; Pastötter et al., 2016; for reviews, see also Bjork, 1998; Sahakyan et al., 2013; Anderson and Hanslmayr, 2014), it is only recently that deliberate selective forgetting has been the subject of investigation (Delaney et al., 2009; Gómez-Ariza et al., 2013; Kliegl et al., 2013; Storm et al., 2013; Aguirre et al., 2014).

A substantial body of research supports the notion that non-selective control can be exerted over episodic memory. Thus, research has shown that we are able to reduce interference from unwanted or no longer relevant memories by making them less accessible (e.g., Geiselman et al., 1983; Bjork, 1989; Bjork and Bjork, 1996; Bäuml et al., 2008; Anderson and Huddleston, 2011; Anderson and Hanslmayr, 2014; for reviews, see also Bjork, 1998; MacLeod, 1998). This phenomenon of motivated forgetting has been widely investigated by using the list-method directed forgetting paradigm (LM-DF; Bjork et al., 1968). In a typical between-subject LM-DF procedure participants firstly study a list of items. Then, half of the participants are cued to forget that list and told to study a second list instead, while the other half is simply told to study the second list of items. Finally, both groups’ memory for Lists 1 and 2 is tested through a free recall test. Results usually show that the group cued to forget exhibits a lower percentage of List 1 recall (a cost effect) than the group cued to keep remembering (Bjork, 1998; MacLeod, 1998), which support the idea that people can intentionally forget. Additionally, the forget group generally exhibit better memory for List 2 items (a benefit effect) than the remember group.

The LM-DF cost has been interpreted from inhibitory and contextual accounts. From the inhibitory view, the cue to forget triggers an inhibitory mechanism that suppresses the subsequent retrieval of the to-be-forgotten (TBF) information, which makes List 1 items less accessible and harder to retrieve in an upcoming memory test. Behavioral (Bjork, 1989; Bjork and Bjork, 1996; Harnishfeger and Pope, 1996; Bjork et al., 1998; Conway et al., 2000; Conway and Fthenaki, 2003; Pastötter and Bäuml, 2007, 2010; Soriano and Bajo, 2007) and neural (Bäuml et al., 2008; Hanslmayr et al., 2012) evidence appear to support this inhibition-based explanation of the LM-DF effect. Thus, for example, it has been shown that List 2 learning modulates List 1 recall so that forgetting is reduced when there is no List 2 to be studied (e.g., Gelfand and Bjork, 1985; Pastötter and Bäuml, 2007) and increases when the number of items of List 2 grows (Pastötter and Bäuml, 2010). On the other hand, the contextual account suggests that the cost of providing an instruction to forget is essentially a context change effect (e.g., Sahakyan and Kelley, 2002). From this view, the cue to forget leads participants to encode List 1 and List 2 as separate events, which produces a mismatch between the study and the test contexts for List 1 in response to the forget instruction. Hence, while List 2 is encoded with the new contextual cues, the retrieval context during the testing of List 1 items mismatches their encoding context, which lowers the recall of these items (for evidence supporting this account see also Sahakyan and Delaney, 2003; Sahakyan et al., 2008; Delaney et al., 2010).

Nevertheless, it is usually the case in everyday situations that people have to select the information that needs updating, and interest has recently sparked in the field of motivated forgetting to investigate the extent to which intentional forgetting may be selective. Delaney et al. (2009) modified the LM-DF procedure to study the ability to voluntarily forget in a selective way. To do so, they had participants study a List 1 consisting of either thematically unrelated or related sentences. Then they asked half of participants to forget just half of List 1 items, which comprised eight sentences about a character named Tom and eight sentences about a character named Alex. Specifically, half of the participants were told to forget the facts learned about Tom and keep remembering the facts learned about Alex. The other half of participants, however, was asked to keep remembering all the sentences about the two characters. Then both groups were presented with a List 2 to study, which was composed of 12 sentences about a third character named Joe. Finally, participants’ memory for List 1 was tested. The result revealed that there was not forgetting in the thematically related condition (which is consistent with the hypothesis that memory for texts and thematically integrated information is better than for unrelated information; e.g., Radvansky, 1999; Gómez-Ariza and Bajo, 2003). More interestingly, however, Delaney et al. (2009) observed a selective directed forgetting (SDF) effect in the thematically unrelated condition; participants cued to forget recalled fewer TBF items (Tom) than did participants cued to remember, while both groups recalled to-be-remembered (TBR) items (Alex) to a similar extent.

The original study by Delaney et al. (2009) has fostered new experiments on selectivity in directed forgetting. Kliegl et al. (2013) also found evidence supporting the idea that LM-DF can be selective. In their Experiment 1, participants studied three lists of unrelated items. After the study of List 2, participants were either asked to keep remembering List 1 and List 2 (remember–remember–remember condition) or to forget List 2 but keep remembering List 1 (remember–forget–remember condition). Both groups studied a third list after remembering or forgetting instructions. Their results showed negative aftereffect of providing a cue to forget: participants in the remember–forget–remember condition showed enhanced recall of the post-cue information (List 3) and less recall of the TBF pre-cue information (List 2) than participants in the remember–remember–remember condition. More important, the forget group did not show poorer recall of the TBR pre-cue information (List 1), which suggests that these participants were able to forget in a selective way. In Experiment 2, they used the same cuing condition as in Experiment 1 (remember–remember–remember and remember–forget–remember) but added a third experimental condition in which study of List 2 was followed by a cue to forget both List 1 and List 2 (forget–forget–remember). The results replicated the main finding of Experiment 1 concerning remember–remember–remember and remember–forget–remember conditions, but also showed that the group cued to forget two lists (forget–forget–remember) had poorer recall of these lists than the other groups. Finally, in a third experiment the authors compared two procedures to selectively cue to forget, namely, the three-list procedure previously used in Experiment 2 and a procedure similar to that used by Delaney et al. (2009) in which the TBR and TBF items were included in the same list (List 1). The results of Experiment 3 showed that both procedures led to comparable forgetting effects. A SDF effect was also found by Gómez-Ariza et al. (2013) in adolescents by using the Delaney et al.’s (2009) procedure. Based on previous research indicating that anxiety entails reduced executive-control capacities (e.g., Bishop, 2009; Pacheco-Unguetti et al., 2010), Gómez-Ariza et al. (2013) aimed to test if this deficit extends to the ability to forget no-longer relevant memories. Their results showed that whereas the healthy control group exhibited a SDF effect, a group diagnosed with social anxiety disorder failed to forget. Finally, Aguirre et al. (2014) replicated the SDF effect in a sample of college students. Following the inhibitory deficit hypothesis (Hasher and Zacks, 1988; Hasher et al., 1999) that claims that older adults suffer from an inhibitory deficit, the researchers aimed to look for the SDF both in young and older adults. While SDF was found in the younger participants, no evidence of selective forgetting was observed in the older ones.

The SDF effect may have important theoretical implications. First, it seems to challenge a general context-change account of directed forgetting effects (Sahakyan and Kelley, 2002). As previously noted, according to this account the LM-DF cost arises because of a mismatch between the study and the retrieval mental context of List 1. This view, however, does not fit well with the SDF effect because the TBF and TBR items are encoded in the same mental context. Hence, it seems odd that only some items from List 1 become forgotten after providing the cue. In contrast, the inhibitory view of LM-DF could in principle account for SDF (Delaney et al., 2009; Gómez-Ariza et al., 2013; Kliegl et al., 2013; Aguirre et al., 2014). As previously described, according to this view (Geiselman et al., 1983; Bjork, 1989, 1998; Conway et al., 2000; Bäuml et al., 2008; Hanslmayr et al., 2012; Anderson and Hanslmayr, 2014) an inhibitory mechanism would make the TBF items (the whole List 1 in a standard DF experiment) less accessible to awareness. Hence, if one assumes that participants in a SDF experiment can encode and segregate the information about the two characters into two different subsets of information, it appears to be possible that, in the presence of a cue to forget, one of the subsets (i.e., Tom items) could uniquely be the target of inhibition in favor of the other subset (i.e., Alex items). Finally, selective rehearsal has also been considered a mechanism to account for SDF (Delaney et al., 2009; Storm et al., 2013; for a rehearsal-based account of the standard DF effect, see Bjork, 1970, 1972). The idea is that after receiving the cue to forget participants would only rehearse the TBR items for the upcoming memory test. Hence, the forgetting of the TBF items would simply result from a lesser amount of processing of these items relative to the items cued to remember.

Whereas both selective rehearsal and inhibition could in principle be the mechanisms underlying SDF, no previous study has directly addressed this issue (for a related work regarding the standard LM-DF procedure, see Pastötter and Bäuml, 2010). In addition, the question of how selectivity is implemented once the forget cue is provided remains unknown. Hence, further research on SDF and its potential modulating factors is necessary. Some pieces of evidence suggest that successfully forget in the standard non-selective LM-DF procedure requires effortful control and draws on executive resources (Harnishfeger and Pope, 1996; Conway et al., 2000; Conway and Fthenaki, 2003; Soriano and Bajo, 2007; Bäuml et al., 2008; Hanslmayr et al., 2012). Similarly, it has been suggested that the ability to selectively intentionally forget taps on executive-control capacities (Gómez-Ariza et al., 2013). However, none of these issues have been directly investigated so far.

The general aims of the present experiments were (1) to better understand SDF by looking at some boundary conditions that could shed light onto the nature of the mechanisms underlying the effect, and (2) to provide evidence that would help to contrast current theoretical accounts of SDF. In Experiment 1, we aimed to replicate the basic effect with the procedure introduced by Delaney et al. (2009) as well as to look at the possible role of rehearsal processes in SDF by using an articulatory suppression task embedded in the SDF procedure. If SDF effect relies on selective rehearsal of the TBR items, then one would expect a significantly reduced SDF effect, if any, under articulatory suppression conditions. In Experiment 2, we explored whether the mechanism underlying SDF relies on controlled processes. By using a dual tasking approach and manipulating the concurrent-task demands, we tried to determine if executive control underpins the capacity to selectively forget. To the extent that SDF depends on attentional control, highly demanding concurrent tasks should prevent SDF. Finally, in Experiment 3, we looked at the selection process in order to explore factors that might constrain SDF. To this end, we manipulated the level of difficulty of the selection process and focus on how successful selective intentional forgetting can be depending on the relative proportion of TBF and TBR information.

Finally, an additional aim of our studies was to replicate SDF effects in standard conditions. Whereas some studies have reported reliable SDF effects (Delaney et al., 2009; Gómez-Ariza et al., 2013; Kliegl et al., 2013; Aguirre et al., 2014), there have been a couple of reported failures to do so (Sahakyan, 2004; Storm et al., 2013). Hence, although the reasons for these discrepancies are not obvious, we thought important to show that SDF is a replicable phenomenon and introduced standard conditions in Experiments 1 and 2.

## Experiment 1

Whereas the SDF effect has been reported in some studies (Delaney et al., 2009; Gómez-Ariza et al., 2013; Kliegl et al., 2013; Aguirre et al., 2014), it has not been always replicated (Sahakyan, 2004; Storm et al., 2013). Furthermore, as previously mentioned, the SDF effect apparently fits well into two different accounts of intentional forgetting. From an inhibitory view (Bjork, 1989; Anderson, 2005; Anderson and Hanslmayr, 2014), SDF could be thought as an aftereffect of an inhibitory-like mechanism in charge of suppressing irrelevant memories (Delaney et al., 2009; Gómez-Ariza et al., 2013; Kliegl et al., 2013; Aguirre et al., 2014). Specifically, the SDF effect could reflect the action of an intentionally driven control mechanism that is selectively targeted to the information cued to forget. Alternatively, SDF could result from a better encoding of the TBR than the TBF items, since participants could engage in selective rehearsal of the former following instructions to selectively forget (Storm et al., 2013). Thus, the aim of the present experiment was twofold. On one hand, our goal was to replicate the SDF effect by using the procedure introduced by Delaney et al. (2009; see also Aguirre et al., 2014). Replicating the effect would entitle us to use our procedure across experiments. On the other hand, we wanted to test the selective rehearsal hypothesis. In this experiment, we used an articulatory suppression task that worked as a concurrent task during List 2 study. In articulatory suppression studies participants are asked to say irrelevant sounds, numbers or syllables while they are memorizing a set of items. The subsequent recall of these items is significantly impaired because of the repetitions, which are thought to prevent items from being rehearsed. From Baddeley’s model of working memory, it has been well established that this concurrent articulation disrupts the action of the phonological loop (Murray, 1968; Baddeley, 1986; Baddeley and Larsen, 2007). Because the articulatory suppression task prevents people from rehearsing (while imposing low attentional demands), it is particularly suitable to address the role of selective rehearsal in producing the basic experimental effect found with the SDF procedure.

A straightforward prediction in the present experiment is that articulatory suppression should reduce the SDF effect to the extent that it relies on rehearsal processes. On the other hand, if SDF does not depend on selective rehearsal, the selective memory cost should even arise when articulatory suppression is performed concurrently. If the latter was the case, one could argue that the mechanism responsible for SDF is independent of rehearsal. That is, the observation of SDF with articulatory suppression as a concurrent task would enable us to claim that SDF is not a byproduct of selective rehearsal.

### Method

#### Participants

One hundred and twelve participants (mean age = 20.46 years; SD = 2.65; 68 women) were randomly assigned to the experimental conditions. All of them were undergraduate students from the University of Granada who received either course credit or money for their participation. This and the following experiments were carried out in accordance with the recommendations of the Research Ethics Committees of the University of Granada, which approved the protocols in advance. All subjects gave written informed consent in accordance with the Declaration of Helsinki (World Medical Association, 2013).

#### Design

The experiment comprised a 2 (condition: standard and articulatory suppression) × 2 (instruction: remember and forget) × 2 (List 1 character: Tom, Alex) mixed design with the latter factor being manipulated within-participants.

#### Materials and Procedure

We used the procedure and the materials developed by Delaney et al. (2009). The lists of items were taken from the unrelated list of sentences used by Delaney et al. (2009). In order to be able to split the whole list of sentences into three subsets to successfully achieve aims for Experiment 3 (see below), two extra sentences were added to List 1 (“Tom/Alex writes in a study” and “Tom/Alex visited Colorado”). List 1 consisted of 18 thematically unrelated sentences about two characters: nine sentences about Tom and nine about Alex (e.g., “Tom watched TV,” “Alex ate a sandwich”). List 2 consisted of 14 sentences about a third character named Joe (e.g., “Joe went online,” “Joe rode a horse”). Both the “subject (Tom/Alex)-predicate” relation and the character to be presented first were counterbalanced across participants, and the sentences of each character were presented in alternated order. Each sentence appeared on the screen for 8 s with an inter item interval of 1 s. In addition, we used a metronome to let participants know the right speed to repeat “Bla.”

**FIGURE 1:**
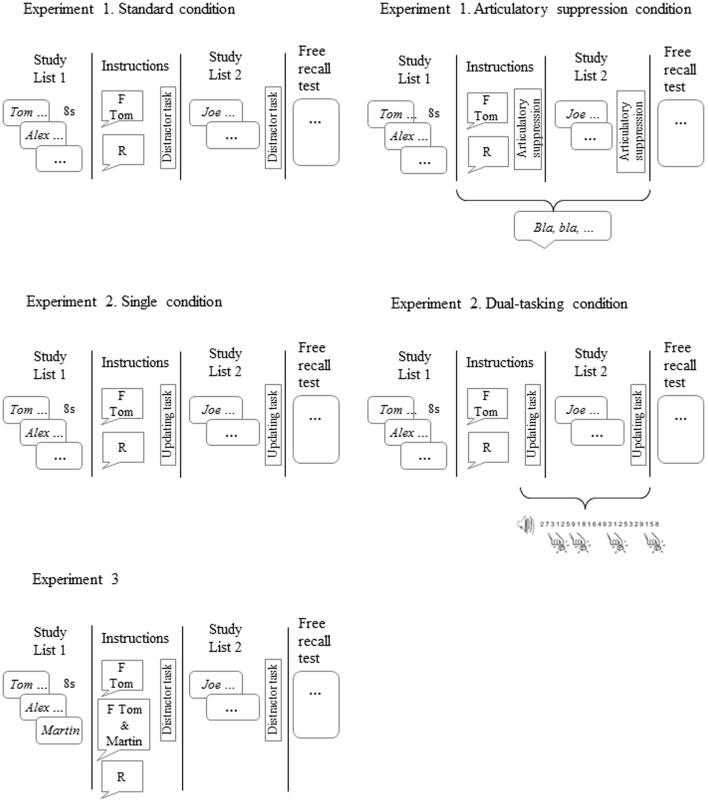
**Experimental procedures**.

Participants were told they would take part in a memory task and were randomly assigned to the “standard” and the “articulatory suppression” conditions. In the “standard” procedure participants were asked to study List 1 sentences. After that, one group (the forget group) was asked to forget the sentences of one character (Tom). They were told the following: “Now you should know that Tom sentences are no longer relevant for the task, so that you are going to be asked only about Alex sentences. Tom sentences were just fillers used to make the memorizing task harder. So in order to have a good performance on the relevant sentences, you should do your best to forget everything about Tom and get rid of the sentences about him. Forgetting this irrelevant information will help you to better recall the information about Alex.” The other group (remember group) was told to keep List 1 in mind. After this, they performed a distracter task consisting on resolving math operations during 90 s, in order to discourage them from rehearsing the material. After that both groups were told to also study List 2. Then they performed again the 90-s distractor task. Following List 2 study, all participants again performed the distractor task for 90 s. Finally both groups were given a sheet of paper to recall as many sentences as possible from List 1 [both Tom (TBF) and Alex (TBR) sentences]. They were given 4 min to do so or until they were done. Then another sheet of paper was given to recall List 2 for the same amount of time. In the “articulatory suppression” condition participants in both remember and forget groups were asked to perform an articulatory task. They were asked to repeatedly say the syllable “Bla” at a rate of 80 beats per minute. Importantly, participants performed articulatory suppression during the intervals of time during which participants would more likely rehearse the TBR items: namely, during List 2 study, just after receiving the forget/remember cue and, also while the instruction was being given. We decided to give participants the remember/forget instruction after they had started the articulatory suppression task because otherwise rehearsal of the TBR items (Alex) could take place while the specific instruction was provided. Thus, by giving the cue to forget while performing the articulatory suppression task we wanted to avoid any chance for the participants to verbally rehearse the TBR material. The procedure took place as follows: first, participants studied List 1 and right after they were asked to perform the articulatory suppression task. Participants started to pronounce “Bla” 10 s before being given any instructions either to forget or remember, and continued with the articulatory suppression task for 90 more seconds. At that point, with participants still saying “Bla,” they were to study List 2 while still engaged in the articulatory suppression task. Once List 2 presentation ended, participants continued saying “Bla” for other 90 s. Once participants in both “standard” and “articulatory suppression” conditions finished the memory test, they were asked to fill out a questionnaire concerning the use of strategies during the study phase. In addition, the group cued to forget was asked to report: (a) whether they really tried to forget the “irrelevant” items when they were cued to do so as well as the reasons behind their behavior; and (b) whether they really believed that their memory for the TBF List 1 character would not be tested later. Importantly, this last question was used as a criterion for replacement of participants who reported not to trust the forget instruction. Although it has been proposed that believing or not the instructions does not affect forgetting in LM-DF experiments (Foster and Sahakyan, 2011; see also Gómez-Ariza et al., 2013; Aguirre et al., 2014), to reduce variability within- and between experiments in the present and following experiments, we replaced participants for non-compliance with the forget instruction (for a similar approach with the think–no think procedure, see Hertel and Calcaterra, 2005; Benoit et al., 2015). Four participants in the “standard” condition and nine in the “articulatory suppression” condition were replaced because they reported they did not believe the forget instruction and that the irrelevant information would not be tested later. The experimental session lasted about 40–45 min. The experimental procedures for the three experiments are shown in **Figure 1**.

### Results

**FIGURE 2:**
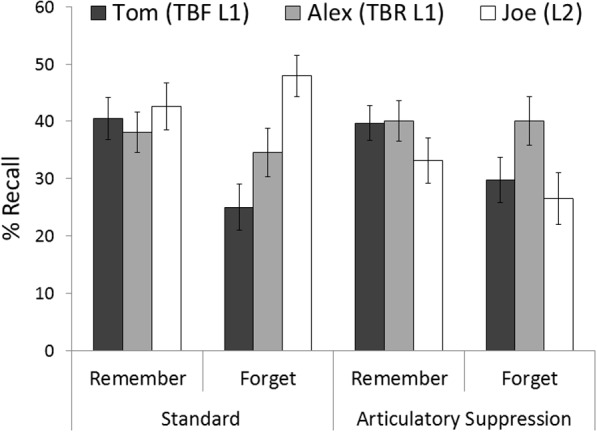
**Mean percentages of correct recall as a function of instruction and character**. Error bars represent standard errors of mean.

In this and the following experiments, recalled items were marked as correct if they kept the gist of the original sentences and contained the studied character–action association (Aguirre et al., 2014). Importantly, because our hypothesis on SDF only concerns List 1, we focus here on reporting performance on this list, even though we also carried out analyses on List 2 recall as complementary measurement of dual tasking. Thus, we first report results from List 1 and then describe analyses for List 2 recall (means and standard errors are shown in **Figure 2**). No analyses were performed in the present work to look at the putative benefit of forgetting List 1 over List 2 recall, since List 2 was always tested the latest and this has been shown to significantly lessen List 2 enhancement (Pastötter et al., 2012).

#### List 1 Recall: Selective Directed Forgetting

We conducted a mixed factorial analysis of variance (ANOVA) on the recall percentages with condition (standard and articulatory suppression) and instruction (remember and forget) as the between-participants factors, and character (Tom and Alex) as the within-participants factor. The ANOVA revealed a main effect of instruction, *F*(1,108) = 4.62, *MSE* = 634.5, *p* < 0.05, $\Omega_{\text{p}}^{2}$ = 0.03 (remember *M* = 39.58, *SD* = 18.13; forget *M* = 32.34, *SD* = 22.14), a main effect of character, *F*(1,108) = 6.4, *MSE* = 174.3, *p* < 0.05, $\Omega_{\text{p}}^{2}$ = 0.04 (Tom *M* = 33.73, *SD* = 20.44; Alex *M* = 38.19, *SD* = 20.43), and a significant interaction between instruction and character, *F*(1,108) = 9.56, *MSE* = 174.3, *p* < 0.01, $\Omega_{\text{p}}^{2}$ = 0.07. The main effect of condition [*F*(1,108) < 1], the interaction condition × instruction [*F*(1,108) < 1] and the interaction character × condition [*F*(1,108) < 1] were not statistically significant. Importantly, because the highest order interaction did not reach statistical significance, [*F*(1,108) < 1, indicating that the performance pattern was comparable in the standard and the articulatory suppression conditions^1^], we further analyzed the significant instruction × character interaction by performing simple effects analyses in each instruction condition. These analyses revealed a reliable SDF effect. While participants cued to forget recalled fewer Tom items than participants cued to remember group, *F*(1,110) = 11.85, *MSE* = 381, *p* < 0.01, $\Omega_{\text{p}}^{2}$ = 0.08, in both instruction conditions participants recalled Alex items to the same extent, *F* < 1.

### List 2 Recall

The ANOVA with condition and instruction as the between-participants factors revealed poorer performance in the articulatory suppression condition (*M* = 29.84; *SD* = 22.55) than in the standard condition (*M* = 45.28; *SD* = 20.39), *F*(1,108) = 14.45, *MSE* = 461.5, *p* < 0.01, *η_p_*^2^ = 0.11, which confirms that participants engaged in articulatory suppression while studying List 2 items. No other effect was reliable [instruction: *F*(1,108) < 1; interaction: *F*(1,108) < 1].

### Discussion

The results of the present experiment reveal a reliable SDF effect in both the standard and the articulatory suppression conditions, which is in line with the findings of some previous studies (Delaney et al., 2009; Gómez-Ariza et al., 2013; Kliegl et al., 2013; Aguirre et al., 2014).

The observation of SDF in the suppression articulatory condition is of special relevance here because it casts doubt on the role that (selective) rehearsal might play in producing the empirical effect. If the SDF effect is, at least in part, a direct consequence of selectively rehearsing the TBR items of List 1, one would expect it to be absent or diminished when rehearsal is prevented by performing articulatory suppression. Although cognitive load is not extreme under articulatory suppression conditions, a number of studies have demonstrated that they prevent information from being rehearsed in working memory (Murray, 1968; Baddeley, 1986; Baddeley and Larsen, 2007). Hence, the fact that SDF is observed in the articulatory suppression condition enables us to suggest that selective rehearsal might not play a key role in producing SDF.

Other aspects of Experiment 1 that do not fit well with the selective rehearsal hypothesis should also be mentioned. First, after being told to either forget or remember, all participants solved a set of math operations that presumably discouraged them from rehearsing. Second, if selective rehearsal were the mechanism responsible for the SDF effect, at least in the standard condition one would expect TBR items to be better recalled in the forget group than in the remember group. This, however, was not the case in this experiment nor was in previous experiments observing SDF (Delaney et al., 2009; Gómez-Ariza et al., 2013; Kliegl et al., 2013; Aguirre et al., 2014).

Whereas the present results do not seem to be in line with a rehearsal-based account of SDF, they could in principle be accounted for by an inhibitory view of directed forgetting (see, for example, Bjork, 1998; Anderson, 2005; Anderson and Hanslmayr, 2014) if one assumes that inhibition can selectively target specific memory traces and it is not affected by articulatory suppression. Importantly, because intentional forgetting is thought to require high levels of attentional control (Conway et al., 2000; Anderson, 2005; Anderson and Hanslmayr, 2014), in Experiment 2, we aimed to better understand the mechanism/s underlying SDF by taking a dual-task approach. According to this methodology, if two tasks rely on the same process, then concurrently performing them will cause a detrimental effect in performance of, at least, one of the tasks. It is important to note that in Experiment 1, we already used a dual-tasking approach. However, because articulatory suppression has been proved not to highly compromise executive control (Murray, 1968; Baddeley, 1986; Baddeley and Larsen, 2007), in Experiment 2, we use a more demanding concurrent task to be performed during List 2 study. Specifically participants studied List 2 while performing an updating task that is well known to rely on executive control (Román et al., 2009; Ortega et al., 2012). To the extent that SDF depends on attentional control after the cue to forget, the effect should be reduced or eliminated with a highly demanding concurrent task.

## Experiment 2

Dual-task procedures have previously been used to study the nature of the mechanisms thought to underlie incidental and intentional types of forgetting. Thus, for example, Román et al. (2009) found that performing a concurrent updating task during retrieval practice led participants to show less retrieval-induced forgetting (an incidental type of forgetting that is thought to be an aftereffect of an inhibitory mechanism that suppresses competing memories during selective retrieval), which suggests that the updating task hindered the executive processes in charge of suppressing competing memories (see also Ortega et al., 2012). As for intentional forgetting, the studies by Conway et al. (2000) and Soriano and Bajo (2007) provide evidence that LM-DF is driven by executive control. In their experiments (Conway et al., 2000, Experiment 4; Soriano and Bajo, 2007, Experiment 2), participants were instructed to learn a sequence of six digits just after receiving the instructions to forget or to remember List 1, and to keep the sequence in mind while learning List 2 since their memory for the digits was going to be tested later. In Conway et al.’s (2000) study (Experiment 4), the memory cost of the instruction to forget was absent in the dual-tasking condition. By using a similar dual-task procedure, Soriano and Bajo (2007; Experiment 2) also failed to observe directed forgetting in a group of participants with low working memory capacity (WMC). Interestingly, they also found that participants with high WMC did show the memory cost even when performing the concurrent task. According to the authors, whereas the concurrent memory task overloaded the cognitive resources of the low WMC participants so that they were not able to exert control over the TBF items in memory, their high WMC counterparts forgot successfully by virtue of a greater availability of executive control resources.

On the basis of the aforementioned results and those from studies on SDF suggesting that selective voluntary forgetting may depend on executive control (Gómez-Ariza et al., 2013; Aguirre et al., 2014), in Experiment 2, we aimed to learn the extent to which the SDF effect may be hindered by overloading attentional control. To do so, we borrowed the continuous updating task used as concurrent task by Román et al. (2009). In this task participants are to listen to a random digits sequence and to press a bottom whenever three odd numbers are presented in a row. Given that this task requires participants to continuously update working-memory contents, it becomes highly demanding and it is especially suited to tax executive control. Thus, and partially following the procedure used in Experiment 1 and in previous dual-tasking studies on intentional forgetting (Conway et al., 2000; Soriano and Bajo, 2007), in the present experiment participants engaged in the updating task just after receiving the remember/forget instruction.

If executive control mediates the SDF effect, overloading control resources with the updating task should compromise the ability to selectively forget. Based on results by Conway et al. (2000) with the standard LM-DF procedure, one could expect the concurrent task to diminish or eliminate the SDF effect, relative to the single condition.

### Method

#### Participants

One hundred and twenty eight participants (mean age = 19.58 years; SD = 2.28; 81 women) were randomly assigned to the experimental conditions. All of them were undergraduate students from the University of Granada who received either course credit or money for their participation. As in Experiment 1, we replaced participants who admitted they did not believe the forget cue and, accordingly, they did not try to forget (nine from in the single condition and two from the dual condition).

#### Design

The experiment comprised a 2 (condition: single and dual-tasking) × 2 (instruction: remember and forget) × 2 (List 1 character: Tom, Alex) mixed design.

#### Materials and Procedure

The details regarding the presentation and the character–action counterbalancing procedures of the experimental sentences were the same as used in Experiment 1. Half of the participants was randomly assigned to the standard “single” SDF condition as introduced by Delaney et al. (2009) and the other half was assigned to perform the “dual-tasking” SDF condition. The single condition closely followed the procedure of the standard condition in Experiment 1, although changes were made in the distracter task to better suit the purpose of the study. Thus, instead of arithmetic operations in this experiment we used an updating task consisting of the auditory presentation of pseudorandom sequences of single digits at a rate of 1 digit per second. The proportion of odd digits was twice the proportion of even digits. Participants were instructed to press a key whenever they heard three odd digits consecutively. We had two reasons to use the updating task as a distracter task. First, and as in our previous experiment, by using a distracter task we minimized the chances to rehearse during the interval between List 1 and List 2 presentation. Second, because participants also performed this task as a concurrent task in the dual-tasking condition, we provided them with practice before introducing the concurrent task during the List 2-study phase. The procedure in the dual-tasking condition was similar to that in the single condition with the only difference that participants were also asked to perform the digit updating task while studying List 2. Once they finished studying List 2, all participants performed the updating task as additional distracter task for 90 s and, finally, they were given the free recall test. Participants filled out the same questionnaire as in Experiment 1.

### Results

**FIGURE 3:**
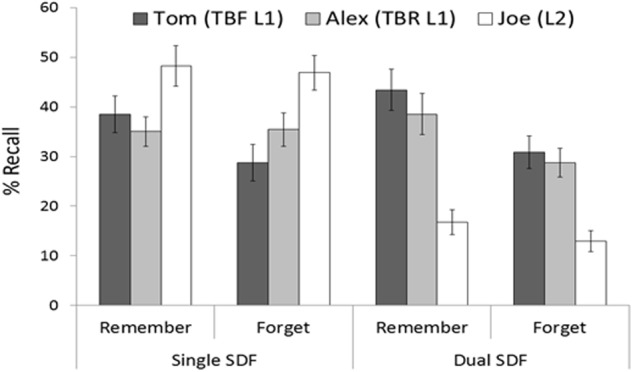
**Mean percentages of correct recall as a function of instruction and character.** Error bars represent standard errors of mean.

As in Experiment 1, we first report results for List 1 and then describe analyses for List 2. Means and standard errors can be seen in **Figure 3**.

#### List 1 Recall: Selective Directed Forgetting

We conducted a mixed factorial ANOVA on the recall percentages with condition (single and dual) and instruction (remember and forget) as the between-participants factors, and character (Tom and Alex) as the within-participants factor. The ANOVA revealed a main effect of instruction, *F*(1,124) = 5.96, *MSE* = 670, *p* < 0.05, $\Omega_{\text{p}}^{2}$ = 0.03 (remember *M* = 38.88, *SD* = 21.23; forget *M* = 30.98, *SD* = 18.79), and a significant interaction between instruction and character, *F*(1,124) = 4.81, *MSE* = 137.1, *p* < 0.05, $\Omega_{\text{p}}^{2}$ = 0.02. The interaction character × condition was marginally significant, *F*(1,124) = 2.95, *MSE* = 137.1, *p* = 0.08, $\Omega_{\text{p}}^{2}$ = 0.01. The main effect of condition [*F*(1,124) < 1], the interaction condition × instruction [*F*(1,124) < 1] and the main effect of character [*F*(1,124) < 1] were not significant. Finally, the highest order interaction did not reach statistical significance, *F*(1,124) = 1.55, *MSE* = 137.1, *p* = 0.21, $\Omega_{\text{p}}^{2}$ = 0.004. Because we expected to replicate the SDF effect in the single condition, to increase statistical power we conducted separated analyses for each condition to gain understanding about if dual tasking prevented selective forgetting.

##### Single condition

The mixed ANOVA with instruction and character as factors showed the interaction to be statistically significant, *F*(1,62) = 6.14, *MSE* = 132, *p* < 0.05, $\Omega_{\text{p}}^{2}$ = 0.07; the simple effects of instruction and character were not [instruction: *F*(1,62) = 1.13, *MSE* = 622.2, *p* = 0.29, $\Omega_{\text{p}}^{2}$ = 0.002; character: *F*(1,62) < 1]. Planned comparisons on the significant interaction revealed that the forget group tended to recall fewer Tom items (TBF items) than the remember group, *F*(1,62) = 3.48, *MSE* = 433.46, *p* = 0.06, $\Omega_{\text{p}}^{2}$ = 0.03, while both groups recalled Alex items (TBR items) to the same extent, *F*(1,62) < 1.

##### Dual condition

Interestingly, there was a significant main effect of instruction, *F*(1,62) = 5.5, *MSE* = 717.8, *p* < 0.05, $\Omega_{\text{p}}^{2}$ = 0.06 (remember group: *M* = 40.97, *SD* = 23.41; forget group: M = 29.86, *SD* = 17.47), thus revealing an overall forgetting effect in the group cued to forget, but no evidence of selectivity. In contrast to the single condition, the ANOVA on the recall percentages indicated that the character × instruction interaction was not reliable, *F*(1,62) < 1. Similarly, the main effect of character [*F*(1,62) = 2.7, *MSE* = 142.1, *p* = 0.10, $\Omega_{\text{p}}^{2}$ = 0.02] failed to reach statistical significance.

#### List 2 Recall

An ANOVA on recall percentages with condition and instruction as the between-participants factors items showed that participants in the dual condition (*M* = 14.84; *SD* = 13.12) recalled fewer items than participants in the single condition (*M* = 47.54; *SD* = 21.22), *F*(1,124) = 108.86, *MSE* = 314.3, *p* < 0.01, $\Omega_{\text{p}}^{2}$ = 0.46. No other source of variance reached statistical significance [instruction: *F*(1,124) < 1; interaction: *F*(1,124) < 1].

##### Updating task

Performance on this task was analyzed by looking at accuracy (errors) and reaction time measures (see **Table 1**). In order to understand the impact of dual tasking on updating performance, we carried out an instruction (remember and forget) × updating condition (first single distracter, dual task, and second single distracter) ANOVA on each dependent measure. The analysis on errors revealed that the only significant source of variance was updating condition, *F*(2,122) = 6.50, *MSE* = 0.0009, *p* < 0.01, $\Omega_{\text{p}}^{2}$ = 0.08 [instruction: *F*(1,122) < 1; interaction instruction × updating condition: *F*(2,122) < 1]. Planned comparisons showed that participants made fewer errors in the first single distracter task than in the dual task condition *F*(1,61) = 12.36, *MSE* = 0.0009, *p* < 0.01, $\Omega_{\text{p}}^{2}$ = 0.15, and the second single distracter task, although this latter effect was only marginal, *F*(1,61) = 3.44, *MSE* = 0.0006, *p* = 0.06, $\Omega_{\text{p}}^{2}$ = 0.03. Similarly, the ANOVA on reaction time (RT) only showed updating condition to be statistically significant, *F*(2,122) = 6.36, *MSE* = 7912, *p* < 0.01, $\Omega_{\text{p}}^{2}$ = 0.07. Planned comparisons revealed that RTs were significantly longer in the dual task [*F*(1,61) = 17.06, *MSE* = 5299.94, *p* < 0.01, $\Omega_{\text{p}}^{2}$ = 0.2] and the second single distracter task [*F*(1,61) = 6.46, *MSE* = 8740.50, *p* < 0.01, $\Omega_{\text{p}}^{2}$ = 0.07] conditions than in the first distracter task condition. These results suggest that updating was impaired by the simultaneous study of List 2 and that single updating after List 2 study was more difficult than single updating before List 2 study.

**Table 1:** Mean error rates and reaction times on the updating task as a function of instruction, dual-tasking and timing.

		Single SDF		Dual SDF	
		Remember	Forget	Remember	Forget
Errors (%)	First single distracter task	0.015 (0.040)	0.018 (0.038)	0.022 (0.059)	0.010 (0.020)
	Concurrent task	–	–	0.041 (0.052)	0.036 (0.047)
	Second single distracter task	0.004 (0.01)	0.039 (0.074)	0.027 (0.043)	0.024 (0.048)
Reaction time (ms)	First single distracter task	681.94 (112.2)	701.68 (131.83)	710.30 (157.13)	705.46 (133.08)
	Concurrent task	–	–	784.42 (166.44)	741.46 (140.41)
	Second single distracter task	705.1 (141.77)	713.84 (118.45)	740.01 (206.79)	764.79 (170.56)

To better understand this last effect, we conducted a second set of analyses in which we considered performance on the updating tasks only when they were performed as distractor tasks in both the dual and single SDF conditions. We first analyzed updating performance before List 2 presentation (1st distractor task). The ANOVA with condition (single and dual) and instruction (remember and forget) as factors showed that for both percentage of errors and RTs there were no reliable effects (all with *F* < 1), indicating that the instruction to forget did not affect updating performance before List 2 was presented.

In contrast, the ANOVA performed on updating errors after List 2 (second distractor task) revealed a significant interaction between condition (single and dual) and instruction (remember and forget), *F*(1,115) = 4.09, *MSE* = 0.002, *p* < 0.05, $\Omega_{\text{p}}^{2}$ = 0.02. Planned comparisons revealed that participants in the forget group of the single SDF condition made significantly more errors than participants in the remember group of the same condition [*F*(1,115) = 6.72, *MSE* = 0.002, *p* < 0.01, $\Omega_{\text{p}}^{2}$ = 0.04], while the forget and remember groups in the dual SDF condition made the same level of errors [*F*(1,115) < 1]. These differences were, however, no evident on RTs (main effects and interactions with *F* < 1). The condition × instruction interaction on errors is interesting because it suggests that the difficulty of updating after List 2 was greater when participants were instructed to forget.

### Discussion

Results from recent studies support the idea that LM-DF effects are driven by inhibitory control (e.g., Bäuml et al., 2008; Hanslmayr et al., 2012). However, the mechanism underlying the SDF effect has not been directly investigated so far. Based on previous results showing that performing a secondary task during List 2 study reduces or eliminates forgetting in standard LM-DF procedures (Conway et al., 2000; Soriano and Bajo, 2007), we used a dual-task methodology with the SDF procedure and added an updating concurrent task during List 2 study. If the mechanism underlying the SDF effect relies on attentional resources, one would not expect forgetting to show up in the dual-tasking condition.

Our results revealed a clearly different pattern of performance as a function of condition. While we replicated the SDF effect in the single condition, the condition with the concurrent updating task during List 2 study exhibited a general forgetting effect of List 1, indicating that dual tasking abolished the selectivity component of the ability to intentionally forget rather than the capacity itself to forget. Hence, the present finding points to an important role of executive control during SDF. While we expected dual tasking to prevent any forgetting from appearing, the observation of overall forgetting of List 1 items suggests that these participants had enough resources available to downregulate memories despite being unable to do it selectively.

Interestingly, the results of the updating task indicated that the group cued to forget in the single SDF condition made more errors than the remember group in the same condition. This difference seems to indicate that when participants are instructed to forget, an effortful executive-control mechanism is triggered during List 2 learning. It is interesting that both the remember and forget groups in the single SDF condition only differ in the instructions received previous to studying List 2 and therefore the increments in errors can only be attributed to the forgetting instructions. One possible reason for this increment in errors might be related to resource depletion caused by the attempts to forget. According to the resource depletion framework, higher cognitive processes are resource limited and can be temporarily exhausted (Engle et al., 1995; Parasuraman, 1998; Muraven and Baumeister, 2000; Anguera et al., 2012). Therefore, the increment in updating errors in the forget condition after List 2 learning might be due to resource depletion due to the executive control processes used during List 2 learning to make part of List 1 less accessible. Regardless the specific mechanism involved in this selective forgetting effect, here we argue that the mechanism recruited by the forget group is cognitively demanding. In addition, the difference in updating performance between the forget and remember conditions was only evident after List 2 learning and it was not present during the updating task performed before presentation of List 2. This suggests that the mechanism leading to forget is not immediately triggered by the instructions to forget, but later on upon presentation of a new list (see Pastötter and Bäuml, 2010).

Taken together, the findings of Experiments 1 and 2 suggest that SDF relies on executive control by revealing differential effects of the two concurrent tasks used in both experiments. Thus, while the updating task seems to hamper the ability to selectively forget, the articulatory suppression task apparently has no effect on the mechanisms underpinning SDF. Hence, it would seem that it is the selection process that is compromised by the increment of executive demands. In order to further understand this process, in the next experiment we attempt to specifically study the role of selectivity in the SDF phenomenon, and how it is implemented, by manipulating the proportion of information to be forgotten and remembered.

## Experiment 3

Experiment 2 showed that overloading attentional control hinders the ability to intentionally forget in a selective way. The aim of the present experiment was to further explore how selectivity is implemented when it comes to intentionally forget. As Kliegl et al. (2013) point out, the issue is not whether directed forgetting may or may not be selective, but under which conditions is, or not, selective. In other words, SDF could be present under some conditions and absent under others. Based on research regarding visual search (Reijnen et al., 2013), we assumed that the proportion of the TBF information (which is supposed to be information to be selected in order to forget it) relative to the TBR information would play a role in the capacity to selectively forget. Thus, in the present experiment we attempt to elucidate if the degree of selectivity demanded by the cue to forget modulates the SDF effect. By doing so, we also expect to learn about the mechanism underlying the effect.

The specific aim of this experiment was twofold. First, we aimed to explore if the degree of selectivity imposed by the task is a factor that determines the presence of SDF. We intended to assess to what extent the memory cost associated with the cue to selectively forget depends upon the amount of TBF items relative to the amount of TBR items. Thus, we test here whether SDF can be observed in a three-subset task by varying the proportion of TBF/TBR items. Second, we aimed to obtain additional evidence that the ability to selectively forget demands executive control.

So far the studies on SDF have used variations of the LM-DF procedure in which people were cued to forget either half of the items of a list (Delaney et al., 2009; Gómez-Ariza et al., 2013; Kliegl et al., 2013; Storm et al., 2013; Aguirre et al., 2014), one out of three lists (Sahakyan, 2004; Kliegl et al., 2013), or two out of three lists (Sahakyan, 2004; Kliegl et al., 2013). However, as far as we know, the proportion of TBF relative to TBR information in the list has never been manipulated. This manipulation would help us to better understand the mechanisms that underlie SDF. In order to correctly accomplish the SDF task, participants need to select the TBF items. Therefore, regardless of the mechanism acting after selection, successful intentional forgetting could be dependent on the relative proportion of information to forget. Because intentional forgetting is thought to require executive control (Conway et al., 2000; Conway and Fthenaki, 2003; Anderson, 2005; Gómez-Ariza et al., 2013; Anderson and Hanslmayr, 2014), and based on our previous results, we argue that the SDF effect could be modulated by the demands of control imposed by the selection process.

To address this issue we modified the standard SDF procedure by including two conditions where an additional character was added to List 1. These conditions were thus composed of three characters so that we were able to manipulate the proportion of List 1 information to be forgotten. Thus, whereas one group was asked to forget one out of three characters, other group was asked to forget two out of three characters. There was also a control group that was asked to remember all three characters. Based on previous research on selective attention using visual search tasks, we expected the TBF/TBR ratio to modulate the SDF effect. Reijnen et al. (2013) found that search efficiency is determined by the target-to-distractor ratio rather than by the absolute difference between the number of distractor and target stimuli. Of relevance for our study, they found an asymmetry effect whereby searching for a smaller number of items among a large set was harder than searching for a larger number among a smaller set of items. Hence, we expected the forget-1/3 condition to involve more difficult selection processes (since participants were to select 33% of the encoded information to forget it), than the forget-2/3 condition (in which participants have to select the 66% of the information). We hypothesized that making SDF more selective could also provide us with some indication regarding whether SDF draws on executive control.

### Method

#### Participants

Seventy-one students from the University of Granada (Spain) participated in the study for either course credits or money (mean age = 22.36 years, *SD* = 4.35; women = 41).

#### Design

The experiment involved a 3 (instruction: remember, forget-1/3 and forget-2/3) × 3(List 1 character: Tom, Alex, and Martin) mixed design, with the latter being within-participants factor.

#### Materials and Procedure

Participants studied two lists of sentences that were created from the lists used in Experiments 1 and 2, but modified to include a third character named Martin. Thus, the original List 1 used in Experiments 1 and 2 containing nine sentences regarding Tom and nine sentences regarding Alex was modified so that it now consisted of six sentences about Tom, six sentences about Alex, and six sentences about Martin. List 2 consisted of the same 14 sentences about the character named Joe. The first character of List 1 to be presented and the action–character assignation were both counterbalanced across participants, resulting in nine versions of the task. The order of presentation of characters was rotated so that each character appeared every three trials. The sentences were presented in the middle of screen for 8 s with 1-s inter-item interval.

The procedure was similar to that used in the standard conditions of Experiments 1 and 2, except for the cues to forget. Thus, in this experiment, one group was cued to forget Tom (forget-1/3 group), another group was told to forget Tom and Martin (forget-2/3 group), and a third group was instructed to remember all characters (remember group). We replaced four participants in the forget-2/3 group because they reported not to believe the instructions to forget.

### Results

#### List 1 Recall: Selective Directed Forgetting

**FIGURE 4:**
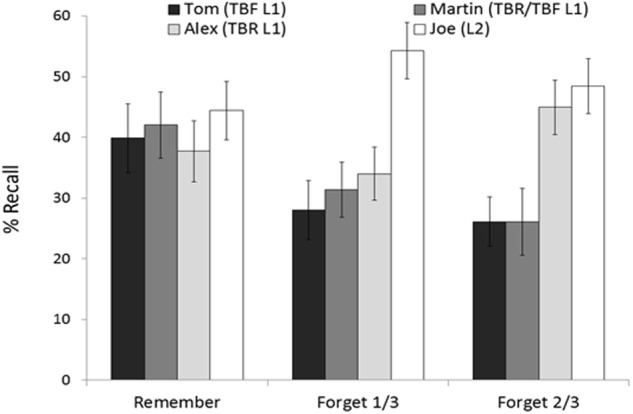
**Mean percentages of correct recall as a function of instruction and character.** Error bars represent standard errors of mean.

We first conducted a mixed factorial ANOVA on recall percentages of List 1, with instruction (remember, forget-1/3, and forget-2/3) as the between-participants factor and character (Tom, Martin, and Alex) as the within-participant factor. The main effect of character, *F*(2,136) = 5.08, *MSE* = 216.5, *p* < 0.01, $\Omega_{\text{p}}^{2}$ = 0.05, and, importantly, the interaction instruction × character were significant *F*(4,136) = 4.55, *MSE* = 216.5, *p* < 0.01, $\Omega_{\text{p}}^{2}$ = 0.09. The main effect of instruction did not reach statistical significance *F*(2,68) = 1.23, *MSE* = 1278.1, *p* = 0.29, $\Omega_{\text{p}}^{2}$ = 0.006. To qualify the interaction, we conducted separate factorial ANOVAs for each forget condition. Means and standard errors can be seen in **Figure 4**.

##### Remember and forget-1/3

The ANOVA 2 (instruction: remember vs. forget-1/3) × 2 (character: Tom, Martin, and Alex) revealed that the interaction was not statistically significant *F*(2,92) < 1. Neither instruction, *F*(1,46) = 2.10, *MSE* = 1302.9, *p* = 0.15, $\Omega_{\text{p}}^{2}$ = 0.02, nor character reached statistical significance, *F*(2,92) < 1.

##### Remember and forget-2/3.

This ANOVA showed a main effect of character, *F*(2,88) = 3.75, *MSE* = 251.3, *p* < 0.05, $\Omega_{\text{p}}^{2}$ = 0.05 (Tom, *M* = 32.97, *SD* = 24.46; Martin, *M* = 34.05, *SD* = 27.20; Alex, *M* = 41.30, *SD* = 22.97), and, more importantly, the interaction instruction × character did reach statistical significance, *F*(2,88) = 7.50, *MSE* = 251.3, *p* < 0.001, $\Omega_{\text{p}}^{2}$ = 0.12. Further analyses revealed a reliable SDF effect: whereas the forget-2/3 group recalled fewer sentences about Tom [*F*(1,44) = 3.87, *MSE* = 562.69, *p* = 0.055, $\Omega_{\text{p}}^{2}$ = 0.05] and Martin [*F*(1,44) = 4.23, *MSE* = 690.60, *p* < 0.05, $\Omega_{\text{p}}^{2}$ = 0.06] than the remember group did, both groups recalled Alex items to the same degree, *F*(1,44) = 1.14, *MSE* = 525.91, *p* = 0.28, $\Omega_{\text{p}}^{2}$ = 0.003. There was a non-significant effect of instruction, *F*(1,44) = 1.51, *MSE* = 1276.5, *p* = 0.22, $\Omega_{\text{p}}^{2}$ = 0.01.

#### List 2 Recall

For completeness, we also carried out a one-way ANOVA on recall percentages of List 2. The results revealed a non-significant main effect of group, *F*(2,68) = 1.15, *MSE* = 513.1, *p* = 0.32, $\Omega_{\text{p}}^{2}$ = 0.004.

### Discussion

The present experiment replicated the SDF effect found in Experiments 1 and 2 (standard and single conditions) and also in some previous studies (Delaney et al., 2009; Gómez-Ariza et al., 2013; Kliegl et al., 2013; Aguirre et al., 2014). Specifically, we observed a clear SDF effect in the low selectivity group (forget-2/3). However, and of relevance for our main purpose, we did not find any evidence of selective forgetting in the condition with the highest demand of selectivity (forget-1/3). Moreover, although this group showed a slight trend toward forgetting the entire List 1, this effect was not reliable indicating that under this more difficult condition participants were not able to use the mechanism that entitle them to forget. This suggests that increments in the difficulty of selection impair the capacity of the participants to select the proper items and to forget.

## Additional Analyses

In order to have a broader picture of the SDF effects observed in the present experiments, we performed additional analyses after collapsing the data of the whole set of participants to increase statistical power. Specifically, we first looked at source confusions rates. Following our previous work on SDF (Aguirre et al., 2014), sentences that originally belonged to Tom (TBF) but participants associated with Alex (TBR) during the recall test (and vice versa) were marked as incorrect and counted as source confusions. Hence, we explored to what extent the poor performance in the present forget conditions was modulated by subject–predicate misattributions. A one-way ANOVA on source confusion rates with instruction (remember, forget, forget-1/3, and forget-2/3) as the factor showed it did not have a reliable effect, *F*(3,307) < 1 (remember: *M* = 0.44, *SD* = 0.77; forget: *M* = 0.58, *SD* = 0.86; forget-1/3: *M* = 0.48, *SD* = 0.82; forget-2/3: *M* = 0.65, *SD* = 0.83). Furthermore, we performed a linear regression analysis with source confusions as the independent variable and an individual SDF index (calculated for each of the participants by subtracting his/her recall rate for the TBF character from the recall rate for the TBR one). This analysis also failed to show a significant effect, *R*^2^ = 0.002, *F*(1,309) < 1, suggesting that source confusions were not responsible for the SDF effect.

We also examined whether output interference could account for the memory impairment for Tom sentences in the forget conditions (see Delaney et al., 2009; Aguirre et al., 2014 for related analyses). If participants in these conditions began to recall with the TBR items more often than did participants in the remember conditions, this could have led them to recall fewer TBF sentences, since output interference would decrease the likelihood of recalling Tom sentences. Thus, we first calculated output position percentiles for each participant (and for each character) according to the method used by Bjork and Whitten (1974). The higher the score, the later the character tended to be recalled. Then, we carried out a regression analysis with the output scores as the predictor variable and the individual SDF index as the dependent variable. The analysis showed that output position did not predict the impairment for Tom sentences relative to Alex sentences, *R*^2^ = 0.001, *F*(1,275) < 1.

Finally, we looked at List 2 performance to examine if, as it has been found with the non-selective LM-DF procedure (Pastötter and Bäuml, 2010; Pastötter et al., 2012), the benefit for List 2 items after the instruction to forget is restricted to early serial positions when List 1 is tested first. Serial position order was simplified by breaking List 2 into three bins (bin 1: items 1–4; bin 2: items 5–9, bin 3: items 10–14). List 2 recall across experiments was analyzed by means of a 4 (instruction: remember, forget-1/2, forget-1/3, forget-2/3) × 3 (serial position: bin 1, bin 2, bin 3) mixed ANOVA. The analysis revealed a main effect of instruction, *F*(3,307) = 8.18, *MSE* = 0.16, *p* < 0.001, $\Omega_{\text{p}}^{2}$ = 0.06. *Post hoc* Tukey tests showed that both forget-1/3 (55%) and forget-2/3 (49%) groups recalled significantly more List 2 items compared to remember (36%) and forget-1/2 (33%) groups (all *p*s < 0.05). No other pairwise comparison was reliable. The omnibus ANOVA also revealed a main effect of serial position, *F*(2,614) = 43.55, *MSE* = 0.04, *p* < 0.001, $\Omega_{\text{p}}^{2}$ = 0.12. Tukey tests showed that the three bins (bin 1 = 55%; bin 2 = 42%; bin 3 = 32%) differed from each other thus confirming an overall primacy effect. There was, however, a reliable interaction instruction × serial position, *F*(6,614) = 3.36, *MSE* = 0.04, *p* < 0.01, $\Omega_{\text{p}}^{2}$ = 0.02. Simple effect analyses and Tukey comparisons confirmed that the benefit for List 2 items of the forget-1/3 and forget-2/3 groups, relative to the remember group, was essentially restricted to bin 1 (although the forget-1/3 group also exhibited better recall than the remember group in bin 2). Hence, the present findings are partially in line with those from previous LM-DF studies showing that when List 1 is tested first, the enhancement of List 2 items that is associated with the instruction to forget only affects the early studied items (Pastötter and Bäuml, 2010; Pastötter et al., 2012). This was the case for the groups forget-1/3 and forget-2/3 of the present study, but it was not when participants were asked to forget half of the List 1. The reasons behind this differential effect are not evident to us, but they might be related to the fact that we did not counterbalanced List 1 and List 2 output order since we were mainly interested on List 1 recall without output interference from List 2. According with Pastötter et al. (2012), List 2 enhancement should mainly arise when List 2 is retrieved before List 1. Future research on SDF should address the issue of List 2 enhancement to gain understanding of the similarities and differences between SDF and standard LM-DF procedures.

## General Discussion

Previous experimental research on selective motivated forgetting has reported mixed results. Thus, whereas some studies have shown SDF effects (Delaney et al., 2009; Gómez-Ariza et al., 2013; Kliegl et al., 2013; Aguirre et al., 2014), others have failed to observe them (Storm et al., 2013; for a study using a related procedure, see Sahakyan, 2004). Hence, and although the reasons behind these divergent findings are not evident, our first aim (essential in order to address the rest of them) was to replicate SDF. The three experiments reported here showed reliable SDF effects (Experiment 1 with the same procedure and population as the Delaney et al.’s study). Therefore, while the mechanism/s responsible for SDF remains unknown, the systematic replication of such an effect across experiments suggests that it is a robust phenomenon.

Experiments 1–3 also aimed to shed light into the cognitive mechanisms underlying SDF. At first sight, SDF does not seem to be easily explained from a general context-change account of directed forgetting. From this perspective, LM-DF effects are a direct consequence of a contextual mismatch between List 1 encoding and retrieval, which is produced by the instruction to forget (Sahakyan and Kelley, 2002; Sahakyan et al., 2013). Although this theory has been successful in accounting for some LM-DF effects, it seems difficult to accommodate to SDF since the TBF and the TBR information is presented within the same context (List 1 study) before receiving the selective cue to forget. Hence, the putative mental context change induced by the cue would be expected to impair the recall of all the items composing List 1. Hence, it is not obvious how SDF could be predictable from a context change account of directed forgetting without requiring additional mechanisms beyond the core ones postulated to date.

However, the SDF effect could be interpreted in terms of both differential rehearsal of forget and remember items or in terms of inhibitory processes acting over the forget items. Although, rehearsal is no longer considered to account for the standard LM-DF effect (e.g., Geiselman et al., 1983; for a review, see Bjork, 1998), in theory, the SDF effect could result from differential encoding for the TBR and TBF sentences of List 1 (see Bjork, 1972, for a similar account of other directed forgetting effects). Thus, the rehearsal account would suggest that after being given the instruction to selectively forget, participants would only rehearse the List 1 items that were cued to remember, which would end up being better encoded than items cued to forget. In Experiment 1, we aimed to explore the role of selectively rehearsing the TBR information in producing SDF. We pursued this goal by introducing an experimental condition whereby sub-vocal rehearsal was putatively disrupted by means of an articulatory suppression secondary task. Despite being a task with low attentional demands, articulatory suppression has shown to be successful at hampering memory encoding in many memory studies (Murray, 1968; Baddeley, 1986; Baddeley and Larsen, 2007). Interestingly, however, Experiment 1 revealed that articulatory suppression did not affect SDF, even though made List 2 less recallable. These findings enable us to claim that differential rehearsal does not seem to play a key role in producing SDF.

In Experiment 2, by using a more conventional dual-task approach we tested the idea that SDF relies on a more active attention-dependent mechanism. From an inhibitory perspective, directed forgetting would be understood as the aftereffect of an inhibition-like mechanism that actively acts upon the studied items after participants receive the cue to forget whenever new material is to be learned (e.g., Geiselman et al., 1983; Bjork, 1998; Pastötter and Bäuml, 2007; Bäuml et al., 2008; Hanslmayr et al., 2012; Anderson and Hanslmayr, 2014). In SDF, the assumption will be that participants encode List 1 items by segregating its two subsets so that inhibition can selectively act on one of them (Delaney et al., 2009; Gómez-Ariza et al., 2013; Kliegl et al., 2013; Aguirre et al., 2014). This putative inhibitory mechanism, therefore, would be contingent on the effective functioning of attentional control mechanisms to first select the TBF information and then to attempt to suppress the selected information from memory. Hence, if the mechanism responsible for SDF involves attentional control, the experimental effect would be smaller or would disappear in the presence of a secondary task entailing executive control. The results of Experiment 2, where the secondary task required participants to update working memory contents (Miyake et al., 2000; for a very similar task, see Román et al., 2009), showed that compromising attentional control right after providing the instruction for selective forgetting affected performance; specifically, participants cued to selectively forget exhibited overall forgetting of List 1 that was not restricted to the TBF items, but equally affected the TBR and TBF items. This finding indicates that it was the selection process, rather than the mechanism in charge of making memories less accessible, that was compromised by the high-demanding secondary task in Experiment 2. This result might in principle seem surprising on the basis of previous studies showing that concurrent tasks abolish (non-selective) directed forgetting (Conway et al., 2000; Soriano and Bajo, 2007). In these studies, the presence of a concurrent task eliminated directed forgetting, whereas in Experiment 2 forgetting was still present. This apparent inconsistency might, however, be explained considering the nature of the concurrent tasks used as well as the demands of the forgetting tasks (selective vs. non-selective). Thus, in the studies by Conway et al. (2000) and Soriano and Bajo (2007), the secondary task was a memory span task wherein participants had to keep six numbers in mind while studying List 2. In contrast, we used a concurrent updating task that made participants to continuously update working memory contents. While it is not obvious to us how these two concurrent tasks might be influencing DF and SDF effects, interactions between updating and selectivity may be producing different results. In addition, Soriano and Bajo (2007) had high and low WM participants in their study and the abolishment of the DF effect with the concurrent task was only observed for the low WM span group. Because we did not assess WMC in our study, it is entirely possible that the presence of a general forgetting effect in Experiment 2 was due to our participants being medium-high WM span participants and, on average, having enough cognitive resources to globally forget, despite being unable to do it selectively (see below for a further consideration of the selection process in SDF). Given that we did not measure WM span in our participants, we are blind regarding how this variable might have influenced our results. Further research regarding WMC and SDF should be conducted to clarify this point. Overall, however, the pattern of results from Experiment 2 seems to indicate that, like other forgetting effects that have been related to memory control (e.g., Conway et al., 2000; Anderson and Levy, 2009; Román et al., 2009; Abel and Bäuml, 2016), SDF is the result of active mechanisms that depend on the availability of attentional resources.

Further support for the idea that SDF recruits executive control comes from the results from Experiment 3, where the TBF/TBR ratio was manipulated and SDF was observed only in the condition where a larger proportion of characters (two out of three) were to be forgotten. Based on previous findings in the realm of visual selective attention (Reijnen et al., 2013), we assumed that correctly performing the SDF task (that is, selecting the forget items and forgetting them) might be harder with low proportion of TBF items. The results of Experiment 3 support this assumption and indicate that the TBF/TBR items ratio modulates the ability to intentionally forget in a selective way. Specifically, it seems that making intentional forgetting more demanding (because of a lower amount of TBF information relative to the TBR information) compromises the selection and downregulation of TBF memories. If so, our finding of no-forgetting effect in the forget-1/3 condition might indirectly suggest that SDF depends on executive control capacities that may be overstressed when the demands for selectivity are high. This interpretation agrees with the results by Gómez-Ariza et al. (2013), who observed a reliable SDF effect in healthy adolescents but failed to do so in a sample of age-matched adolescents diagnosed with social anxiety. Gómez-Ariza et al. (2013) attributed the lack of forgetting in the clinical sample to the executive control deficits associated with high anxiety and suggested that SDF involves executive control.

Besides indicating that SDF is modulated by the selectivity demands imposed by the task, which might be indicative of the role that executive control might play in SDF, the results of Experiment 3 join those of Experiment 2 to stress the role of selection in SDF. The pattern of results in these two experiments indicated that increasing selection demands was more disruptive (Experiment 3; no general forgetting effect) than increments in attentional resources produced by dual task performance (Experiment 2; generalized forgetting effect, but not selective). Thus, in Experiment 2 selectivity but not forgetting was affected whereas in Experiment 3 both selection and forgetting processes were harmed by the experimental manipulation. Although it is not obvious why increments in selection seem to be more disruptive than dual tasking, the obtained pattern of results seems to suggest that at least two different processes (selection and forgetting) underpin the ability to intentionally forget in a selective way.

Then, what does exactly produce the selective memory impairment observed in the SDF paradigm? Although the present experiments do not provide direct evidence for specific mechanisms, altogether the present findings favor the idea that SDF is a consequence of active attention-driven mechanisms. The results of the three experiments reported here add to previous SDF findings (Gómez-Ariza et al., 2013; Kliegl et al., 2013; Aguirre et al., 2014) to support the idea that motivated forgetting (even when it is selective) relies on goal-oriented executive-control mechanisms (Geiselman et al., 1983; Anderson, 2005; Bäuml et al., 2008; Hanslmayr et al., 2012; Anderson and Hanslmayr, 2014). Specifically, an inhibitory mechanism associated with activity in prefrontal areas has been proposed to be in charge of making episodic memories temporary less accessible (Bäuml et al., 2008; Hanslmayr et al., 2012; for a review, see Anderson and Hanslmayr, 2014). Bäuml et al. (2008), using electroencephalography and oscillation analyses, found that the instruction to forget decreased the large-scale synchrony in a widespread cortical network that seems to be involved in memory retention. Hanslmayr et al. (2012) replicated these results and interestingly found that repetitive transcranial magnetic stimulation of the dorsolateral prefrontal cortex reduced neural synchrony and significantly increased the effect of directed forgetting. More recently, it has been shown that suppressing cortical activity in the right lateral prefrontal cortex (by means of transcranial direct current stimulation) abolishes the directed forgetting effect (Silas and Brandt, 2016). Although we do not have direct evidence that inhibitory processes underlie SDF, the global pattern is consistent with an account in which selection and inhibition of TBF items is responsible for the obtained SDF effects. Future studies should attempt to provide direct evidence of the specific mechanism underling the effect.

## Author Contributions

This work is part of the thesis dissertation of the first author as part of the doctoral work for the Doctoral Program in Psychology at the University of Granada. CA, CG-A, and MB developed the concept and the design of the three experiments together. CA contributed to the implementation of the tasks, data collection and analyses, and manuscript writing. PA and GM contributed to the planning and design of the third experiment and contributed to the writing. MB and CG-A jointly supervised the processes of accomplishing the study, writing, reviewing, and approving the final version of the manuscript.

## Conflict of Interest Statement

The authors declare that the research was conducted in the absence of any commercial or financial relationships that could be construed as a potential conflict of interest.

## Footnotes

1 In fact, the interaction instruction × character showed to be statistically significant and comparable in effect sizes in both conditions [standard condition: *F*(1,54) = 4.29, *MSE* = 231, *p* < 0.05, $\Omega_{\text{p}}^{2}$ = 0.05; articulatory suppression condition: *F*(1,54) = 5.86, *MSE* = 117, *p* < 0.05, $\Omega_{\text{p}}^{2}$ = 0.07].

## References

[B1] Abel M. Bäuml K. H. (2016). Retrieval practice can eliminate list-method directed forgetting. *Mem. Cognit.* 44 15–23. 10.3758/s13421-015-0539-x 26286882

[B2] Aguirre C. Gómez-Ariza C. J. Bajo M. T. Andrés P. Mazzoni G. (2014). Selective voluntary forgetting in young and older adults. *Psychol. Aging* 29 128–139. 10.1037/a0035598 24660801

[B3] Anderson M. C. (2005). “The role of inhibitory control in forgetting unwanted memories: a consideration of three methods,” in *Dynamic Cognitive Processes*, eds MacLeod C. Uttl B. (Tokyo: Springer-Verlag), 159–190.

[B4] Anderson M. C. Hanslmayr S. (2014). Neural mechanisms of motivated forgetting. *Trends Cogn. Sci.* 18 279–292. 10.1016/j.tics.2014.03.002 24747000 PMC4045208

[B5] Anderson M. C. Huddleston E. (2011). Towards a cognitive and neurobiological model of motivated forgetting. *Nebr. Symp. Motiv.* 58 53–120. 10.1007/978-1-4614-1195-6_3 22303764

[B6] Anderson M. C. Levy B. J. (2009). Suppressing unwanted memories. *Curr. Dir. Psychol. Sci.* 18 184–194. 10.1111/j.1467-8721.2009.01634.x PMC5390940 28458471

[B7] Anguera J. A. Bernard J. A. Jaeggi S. M. Buschkuehl M. Benson B. L. Jennett S. (2012). The effects of working memory resource depletion and training on sensorimotor adaptation. *Behav. Brain Res.* 228 107–115. 10.1016/j.bbr.2011.11.040 22155489 PMC3264800

[B8] Baddeley A. D. (1986). *Working Memory.* New York, NY: Oxford University Press.

[B9] Baddeley A. D. Larsen J. D. (2007). The phonological loop: some answers and some questions. *Q. J. Exp. Psychol.* 60 512–518. 10.1080/17470210601147663

[B10] Bäuml K.-H. Hanslmayr S. Pastötter B. Klimesch W. (2008). Oscillatory correlates of intentional updating in episodic memory. *Neuroimage* 41 596–604. 10.1016/j.neuroimage.2008.02.053 18420423

[B11] Benoit R. G. Hulbert J. C. Huddleston E. Anderson M. C. (2015). Adaptive top-down suppression of hippocampal activity and the purging of intrusive memories from consciousness. *J. Cogn. Neurosci.* 27 96–111. 10.1162/jocn_a_00696 25100219

[B12] Bishop S. J. (2009). Trait anxiety and impoverished prefrontal control of attention. *Nat. Neurosci.* 12 92–98. 10.1038/nn.2242 19079249

[B13] Bjork E. L. Bjork R. A. (1996). Continuing influences of to-be forgotten information. *Conscious. Cogn.* 5 176–196. 10.1006/ccog.1996.0011 8978530

[B14] Bjork E. L. Bjork R. A. Anderson M. C. (1998). “Varieties of goal-directed forgetting,” in *Intentional Forgetting: Interdisciplinary Approaches*, eds Golding J. M. MacLeod C. M. (Mahwah NJ: L. Erlbaum Associates), 103–137.

[B15] Bjork R. A. (1970). Positive forgetting: the noninterference of items intentionally forgotten. *J. Verbal Learn. Verbal Behav.* 98 255–268. 10.1016/S0022-5371(70)80059-7

[B16] Bjork R. A. (1972). “Theoretical implications of directed forgetting,” in *Coding Processes in Human Memory*, eds Melton A. W. Martin E. (Washington, DC: Winston), 217–235.

[B17] Bjork R. A. (1989). “Retrieval inhibition as an adaptive mechanism in human memory,” in *Varieties of Memory and Consciousness: Essays in Honour of Endel Tulving*, eds Roediger H. L. Craik F. I. M. (Hillsdale: NJ: Erlbaum), 309–330.

[B18] Bjork R. A. (1998). “Intentional forgetting in perspective: comments, conjectures, and some directed remembering,” in *Intentional Forgetting: Interdisciplinary Approaches*, eds Golding J. M. MacLeod C. M. (Hillsdale, NJ: Erlbaum), 453–481.

[B19] Bjork R. A. LaBerge D. LeGrand R. (1968). The modification of short-term memory through instructions to forget. *Psychon. Sci.* 10 55–56. 10.3758/BF03331404

[B20] Bjork R. A. Whitten W. B. (1974). Recency-sensitive retrieval processes in long-term free recall. *Cogn. Psychol.* 6 173–189. 10.1016/j.neuropsychologia.2009.02.013

[B21] Conway M. A. Fthenaki A. (2003). Disruption of inhibitory control of memory following lesions to the frontal and temporal lobes. *Cortex* 39 667–686. 10.1016/S0010-9452(08)70859-1 14584548

[B22] Conway M. A. Harries K. Noyes J. Racsmány M. Frankish C. (2000). The disruption and dissolution of directed forgetting: inhibitory control of memory. *J. Mem. Lang.* 43 409–430. 10.1006/jmla.2000.2706

[B23] Delaney P. F. Nghiem K. N. Waldum E. R. (2009). The selective directed forgetting effect: can people forget only part of a text? *Q. J. Exp. Psychol.* 62 1542–1550. 10.1080/17470210902770049 19370484

[B24] Delaney P. F. Sahakyan L. Kelley C. M. Zimmerman C. A. (2010). Remembering to forget: the amnesic effect of daydreaming. *Psychol. Sci.* 2 1036–1042. 10.1177/0956797610374739 20548055

[B25] Engle R. W. Conway A. R. A. Tuholski S. W. Shisler R. J. (1995). A resource account of inhibition. *Psychol. Sci.* 6 122–125. 10.1111/j.1467-9280.1995.tb00318.x

[B26] Foster N. L. Sahakyan L. (2011). The role of forget-cue salience in list-method directed forgetting. *Memory* 19 110–117. 10.1080/09658211.2010.537665 21240753

[B27] Geiselman R. E. Bjork R. A. Fishman D. (1983). Disrupted retrieval in directed forgetting: a link with posthypnotic amnesia. *J. Exp. Psychol. Gen.* 112 58–72. 10.1080/09658210244000090 6221062

[B28] Gelfand H. Bjork R. A. (1985). “On the locus of retrieval inhibition in directed forgetting,” in *Paper Presented at the Psychonomics Society*, Boston, MA.

[B29] Gómez-Ariza C. J. Bajo M. T. (2003). Interference and integration: the fan effect in children and adults. *Mem. Cogn.* 11 505–523. 10.1080/09658210244000090 14982119

[B30] Gómez-Ariza C. J. Iglesias-Parro S. García-Lopez L. J. Díaz-Castela M. Espinosa-Fernández L. Muela J. A. (2013). Selective intentional forgetting in adolescents with social anxiety disorder. *Psychiatry Res.* 208 151–155. 10.1016/j.psychres.2012.09.027 23068080

[B31] Hanslmayr S. Volberg G. Wimber M. Oehler N. Staudigl T. Hartmann T. (2012). Prefrontally driven down-regulation of neural synchrony mediates goal-directed forgetting. *J. Neurosci.* 32 14742–14751. 10.1523/JNEUROSCI.1777-12.2012 23077059 PMC6621432

[B32] Harnishfeger K. K. Pope R. S. (1996). Intending to forget: the development of cognitive inhibition in directed forgetting. *J. Exp. Child Psychol.* 62 292–315. 10.1006/jecp.1996.0032 8683190

[B33] Hasher L. Zacks R. T. (1988). Working memory, comprehension, and aging: a review and a new view. *Psychol. Learn. Motiv.* 22 193–225. 10.1016/S0079-7421(08)60041-9

[B34] Hasher L. Zacks R. T. May C. P. (1999). “Inhibitory control, circadian arousal, and age,” in *Attention & Performance, XVII, Cognitive Regulation of Performance: Interaction of Theory and Application*, eds Gopher D. Koriat A. (Cambridge: MA: MIT Press), 653–675.

[B35] Hertel P. T. Calcaterra G. (2005). Intentional forgetting benefits from thought substitution. *Psychon. Bull. Rev.* 12 484–489. 10.3758/BF03193792 16235633

[B36] Kliegl O. Pastötter B. Bäuml K.-H. T. (2013). List-method directed forgetting can be selective: evidence from the 3-list and the 2-list tasks. *Mem. Cogn.* 41 452–464. 10.3758/s13421-012-0264-7 23132519

[B37] MacLeod C. (1998). “Directed forgetting,” in *Intentional Forgetting: Interdisciplinary Approaches*, eds Golding J. M. MacLeod C. (Hillsdale: NJ: Erlbaum), 157.

[B38] Miyake A. Friedman N. P. Emerson M. J. Witzki A. H. Howerter A. Wager T. (2000). The unity and diversity of executive functions and their contributions to complex “frontal lobe” tasks: a latent variable analysis. *Cogn. Psychol.* 41 49–100. 10.1006/cogp.1999.0734 10945922

[B39] Muraven M. Baumeister R. F. (2000). Self-regulation and depletion of limited resources: does self-control resemble a muscle. *Psychol. Bull.* 126 247–259. 10.1037/0033-2909.126.2.247 10748642

[B40] Murray D. J. (1968). Articulation and acoustic confusability in short-term memory. *J. Exp. Psychol. Gen.* 78 679–684. 10.1037/h0026641

[B41] Ortega A. Gómez-Ariza C. J. Román P. E. Bajo M. T. (2012). Memory inhibition, aging and the executive deficit hypothesis. *J. Exp. Psychol. Learn. Mem. Cogn.* 38 178–186. 10.1037/a0024510 21767066

[B42] Pacheco-Unguetti A. P. Acosta A. Callejas A. Lupiáñez J. (2010). Attention and anxiety: different attentional functioning under state and trait anxiety. *Psychol. Sci.* 21 298–304. 10.1177/0956797609359624 20424060

[B43] Parasuraman R. (ed.) (1998). *The Attentive Brain.* Cambridge: MIT Press.

[B44] Pastötter B. Bäuml K. H. (2007). The crucial role of postcue encoding in directed forgetting and context-dependent forgetting. *J. Exp. Psychol. Learn. Mem. Cogn.* 33 977–982. 10.1037/0278-7393.33.5.977 17723074

[B45] Pastötter B. Bäuml K. H. (2010). Amount of postcue encoding predicts amount of directed forgetting. *J. Exp. Psychol. Learn. Mem. Cogn.* 36 54–65. 10.1037/a0017406 20053044

[B46] Pastötter B. Kliegl O. Bäuml K. H. (2012). List-method directed forgetting: the forget cue improves both encoding and retrieval of postcue information. *Mem. Cogn.* 40 861–873. 10.3758/s13421-012-0206-4 22588948

[B47] Pastötter B. Kliegl O. Bäuml K. H. (2016). List-method directed forgetting: evidence for the reset-of-encoding hypothesis employing item-recognition testing. *Mem. Cogn.* 24 63–74. 10.1080/09658211.2014.985589 25483244

[B48] Radvansky G. A. (1999). Memory retrieval and suppression: the inhibition of situation models. *J. Exp. Psychol. Gen.* 128 563–579. 10.1037/0096-3445.128.4.563 10650586

[B49] Reijnen E. Wolfe J. M. Krummenacher J. (2013). Coarse guidance by numerosity in visual search. *Atten. Percept. Psychophys.* 75 16–28. 10.3758/s13414-012-0379-8 23070885 PMC4037405

[B50] Román P. E. Soriano M. F. Gómez-Ariza C. J. Bajo M. T. (2009). Retrieval-induced forgetting and executive control. *Psychol. Sci.* 20 1053–1058. 10.1111/j.1467-9280.2009.02415.x 19656337

[B51] Sahakyan L. (2004). Destructive effects of “forget” instructions. *Psychon. Bull. Rev.* 11 555–559. 10.3758/BF03196610 15376810

[B52] Sahakyan L. Delaney P. F. (2003). Can encoding differences explain the benefits of directed forgetting in the list-method paradigm? *J. Mem. Lang.* 48 195–201. 10.1016/S0749-596X(02)00524-7

[B53] Sahakyan L. Delaney P. F. Foster N. L. Abushanab B. (2013). “List method directed forgetting in cognitive and clinical research: a theoretical and methodological review,” in *The Psychology of Learning and Motivation*, ed. Ross B. H. (Philadelphia, PA: Elsevier), 131–189. 10.1016/b978-0-12-407187-2.00004-6

[B54] Sahakyan L. Delaney P. F. Goodmon L. B. (2008). “Oh, honey, I already forgot that”: strategic control of directed forgetting in older and younger adults. *Psychol. Aging* 23 621–633. 10.1037/a0012766 18808251 PMC2562336

[B55] Sahakyan L. Kelley C. M. (2002). A contextual change account of the directed forgetting effect. *J. Exp. Psychol. Learn. Mem. Cogn.* 28 1064–1072. 10.1037/0278-7393.28.6.1064 12450332

[B56] Silas J. Brandt K. R. (2016). Frontal transcranial direct current stimulation (tDCS) abolishes list-method directed forgetting. *Neurosci. Lett.* 11 166–169. 10.1016/j.neulet.2016.01.035 26820374

[B57] Soriano M. F. Bajo M. T. (2007). Working memory resources and interference in directed forgetting. *Psicologica* 28 63–85.

[B58] Storm B. C. Koppel R. H. Wilson B. M. (2013). Selective cues to forget can fail to cause forgetting. *Q. J. Exp. Psychol.* 66 29–36. 10.1080/17470218.2012.753923 23281848

[B59] World Medical Association (2013). World medical association declaration of helsinki: ethical principles for medical research involving human subjects. *JAMA* 310 2191–2194. 10.1001/jama.2013.281053 24141714

